# Transcriptomic analysis highlights cochlear inflammation associated with age-related hearing loss in C57BL/6 mice using next generation sequencing

**DOI:** 10.7717/peerj.9737

**Published:** 2020-08-19

**Authors:** Zhongwu Su, Hao Xiong, Yi Liu, Jiaqi Pang, Hanqing Lin, Weijian Zhang, Yiqing Zheng

**Affiliations:** 1 Department of Otolaryngology, Sun Yat-sen Memorial Hospital, Sun Yat-sen University, Guangzhou, China; 2 Institute of Hearing and Speech-Language Science, Sun Yat-sen University, Guangzhou, China; 3 Department of Otolaryngology, Guangdong Women and Children Hospital, Guangzhou, China

**Keywords:** Age-related hearing loss, Inflammation, Aging, Apoptosis, Necroptosis, RNA sequencing

## Abstract

**Background:**

In our aging society, age-related hearing loss (AHL) is the most common sensory disorder in old people. Much progress has been made in understanding the pathological process of AHL over the past few decades. However, the mechanism of cochlear degeneration during aging is still not fully understood.

**Methods:**

Next generation sequencing technique was used to sequence the whole transcriptome of the cochlea of C57BL/6 mice, a mouse model of AHL. Differentially expressed genes (DEGs) were identified using the Cuffdiff software. GO and KEGG pathway enrichment analyses of the DEGs were implemented by using the GOseq R package and KOBAS software, respectively.

**Results:**

A total of 731 genes (379 up- and 352 down-regulated) were revealed to be differentially expressed in the cochlea of aged mice compared to the young. Many genes associated with aging, apoptosis, necroptosis and particularly, inflammation were identified as being significantly modulated in the aged cochlea. GO and KEGG analyses of the upregulated DEGs revealed that the most enriched terms were associated with immune responses and inflammatory pathways, whereas many of the downregulated genes are involved in ion channel function and neuronal signaling. Real-time qPCR showed that H_2_O_2_ treatment significantly induced the expression of multiple inflammation and necroptosis-related genes in HEI-OC1 cells.

**Conclusion:**

Using next generation sequencing, our transcriptomic analysis revealed the differences of gene expression pattern with age in the cochlea of C57BL/6 mice. Our study also revealed multiple immune and inflammatory transcriptomic changes during cochlear aging and provides new insights into the molecular mechanisms underlying cochlear inflammation in AHL.

## Introduction

Age-related hearing loss (AHL) or presbycusis is a universal sensory disorder in modern society and affects about 25–40% of people over 65 years (Tavanai & Mohammadkhani, 2017; Wang & Puel, 2020; Yamasoba et al., 2013). Hearing loss significantly affects the daily communication and contributes to social isolation, depression and possibly dementia. Multiple factors such as genetic predisposition, epigenetic factors, aging and noise or ototoxic drug exposure are the causes of AHL (Tavanai & Mohammadkhani, 2017; Wang & Puel, 2020; Yamasoba et al., 2013). The underlying mechanisms of AHL include oxidative stress, mitochondrial DNA mutations, autophagy impairment and non-coding RNA disorders (Tavanai & Mohammadkhani, 2017; Wang & Puel, 2020; Yamasoba et al., 2013; Su et al., 2019). However, the mechanism of cochlear degeneration during aging is still not fully understood.

In recent years, the effects of inflammation on aging-related disorders have been extensively investigated. During aging, the body suffers from chronic low-grade inflammation, a phenomenon also referred to as “inflammaging”. Chronic inflammation is a consequence of immunosenescence, the aging of the immune system, and is primarily characterized by increased levels of proinflammatory cytokines in response to various stressors (Watson et al., 2017; Baylis et al., 2013). Compelling evidence suggests that chronic inflammation contributes critically to the initiation and progression of multiple age-related diseases, including neurodegeneration disease, cardiovascular disease and type-II diabetes (Pawelec, Goldeck & Derhovanessian, 2014; Chen & Xu, 2015). However, only little research on the potential role of inflammation in AHL has been reported. Verschuur et al. (2012) reported that markers of inflammatory status including white blood cell count, neutrophil count, IL-6 and C-reactive protein were significantly associated with the degree of hearing loss in older people. The same group also revealed a similar observation on a different population data set (Verschuur, Agyemang-Prempeh & Newman, 2014). Their studies uncovered a possible causal link between systemic inflammatory status and hearing loss in olderly people. In addition, several studies observed the population and morphology changes of macrophages with age in human and mouse cochleae (Noble et al., 2019; Frye et al., 2017). These findings indicate the possible involvement of macrophage activation in age-related cochlear degeneration. Iwai & Inaba (2015) reported that the rejuvenation of systemic immune function by fetal thymus grafts helped to improve AHL in SAMP1 mice.

Using next generation sequencing, our current study aimed to reveal transcriptomic alterations during aging in the cochlea of C57BL/6 mice. Bioinformatics analysis was further conducted to uncover biological processes and pathways associated with AHL.

## Materials and Methods

### Cell culture

Auditory cell line HEI-OC1 cells were a gift from F. Kalinec (the House Ear Institute, Los Angeles, CA, USA) and were cultured in high-glucose DMEM (Gibco, Gaithersburg, MD, USA) with 10% fetal bovine serum (Gibco, Gaithersburg, MD, USA) at 33 °C in an incubator containing 10% CO_2_. HEI-OC1 cells were treated with 500 μM H_2_O_2_ (Sigma–Aldrich, St. Louis, MO, USA) for 24 h and then collected for RNA extraction.

### Animals

C57BL/6 mice were obtained from the Laboratory Animal Center of Sun Yat-sen University (Guangzhou, China). The experimental groups consisted of 4-week old and 12-month old mice (*n* = 9/group). Mice used in this study are part of the animals used in our previous study and were all examined by auditory brainstem response tests (Su et al., 2019). All animals were housed in pathogen-free facilities with access to food and water ad libitum. All experiments were performed according to the protocols approved by the Institutional Animal Care and Use Committee (IACUC), Sun Yat-sen University (Approval No.: SYSU-IACUC-2017-B0034).

### Tissue preparations

The procedure for cochlear tissue preparations was followed as previously described (Su et al., 2019). Briefly, mice were anesthetized with an intraperitoneal injection of a mixture of ketamine (100 mg/kg) and xylazine (10 mg/kg). The deeply anesthetized mice were decapitated and cochleae were extracted from the temporal bones of the mice. The bony capsule and modiolus were removed under a dissection microscope.

### RNA-sequencing data

We previously conducted RNA-sequencing with cochleae of C57BL/6 mice using next generation sequencing, and reported the alteration of long non-coding RNA (lncRNA) in the cochlea with age (Su et al., 2019). The sequencing data were submitted to the NCBI Gene Expression Omnibus (GEO) under accession number GSE127204. In the previous study, data was generated from the lncRNA sequencing libraries. The sequencing data were used for mRNA expression analysis in this study.

### Differential expression analysis

The raw data was processed as previously described (Su et al., 2019). Differentially expressed transcripts were identified using the Cuffdiff software. Transcripts with *p*-adjust < 0.05, |log2 (fold change)| > 1 were defined as differentially expressed genes (DEGs).

### Gene ontology and KEGG pathway enrichment analysis

Gene ontology (GO) enrichment analysis of the DEGs was conducted by using the GOseq R package. GO terms with *p*-adjust < 0.05 were considered significantly enriched. Kyoto Encyclopedia of Genes and Genomes (KEGG) pathway enrichment analysis of the DEGs was implemented by using the KOBAS software.

### RNA extraction and quantitative real-time PCR

Total RNA was extracted from cochlear tissues or HEI-OC1 cells using TRIzol reagent (Invitrogen, Waltham, MA, USA) as directed by the manufacturer. The cochleae of three mice were pooled for RNA extration. Extracted RNA was reverse transcribed into cDNA with PrimeScript RT Master Mix (Takara, Shiga, Japan). The real-time qPCR assays were carried out on a Roche LightCycler 96 real-time PCR system (Roche, Basel, Switzerland) with TB Green Premix Ex Taq II (Takara, Shiga, Japan). Primer sequences used in this study were as follows: *CCL2* forward: 5′-TTAAAAACCTGGATCGGAACCAA-3′, reverse: 5′-GCATTAGCTTCAGATTTACGGGT-3′; *CXCL13* forward: 5′-GGCCACGGTATTCTGGAAGC-3′, reverse: 5′-GGGCGTAACTTGAATCCGATCTA-3′; *IRF7* forward: 5′-GAGACTGGCTATTGGGGGAG-3′, reverse: 5′-GACCGAAATGCTTCCAGGG-3′; *ZBP1* forward: 5′-CTCCTGCAATCCCTGAGAACT-3′, reverse: 5′-GGCTACATGGCAAGACTATGTC-3′; *ALOX15* forward: 5′-GGCTCCAACAACGAGGTCTAC-3′, reverse: 5′-AGGTATTCTGACACATCCACCTT-3′; *CEBPB* forward: 5′-AAGCTGAGCGACGAGTACAAGA-3′, reverse: 5′-GTCAGCTCCAGCACCTTGTG-3′; *FOS* forward: 5′-CGGGTTTCAACGCCGACTA-3′, reverse: 5′-TTGGCACTAGAGACGGACAGA-3′; *MLKL* forward: 5′-AATTGTACTCTGGGAAATTGCCA-3′, reverse: 5′-AAAGACTCCTACCGTCCACAG-3′; *GAPDH*: forward: 5′-GGTCATCCATGACAACTTTGG-3′, reverse: 5′-GGCCATCACGCCACAG-3′.

## Results

### Differential expression profile of genes in the cochlea of aged C57BL/6 mice

**Figure 1:**
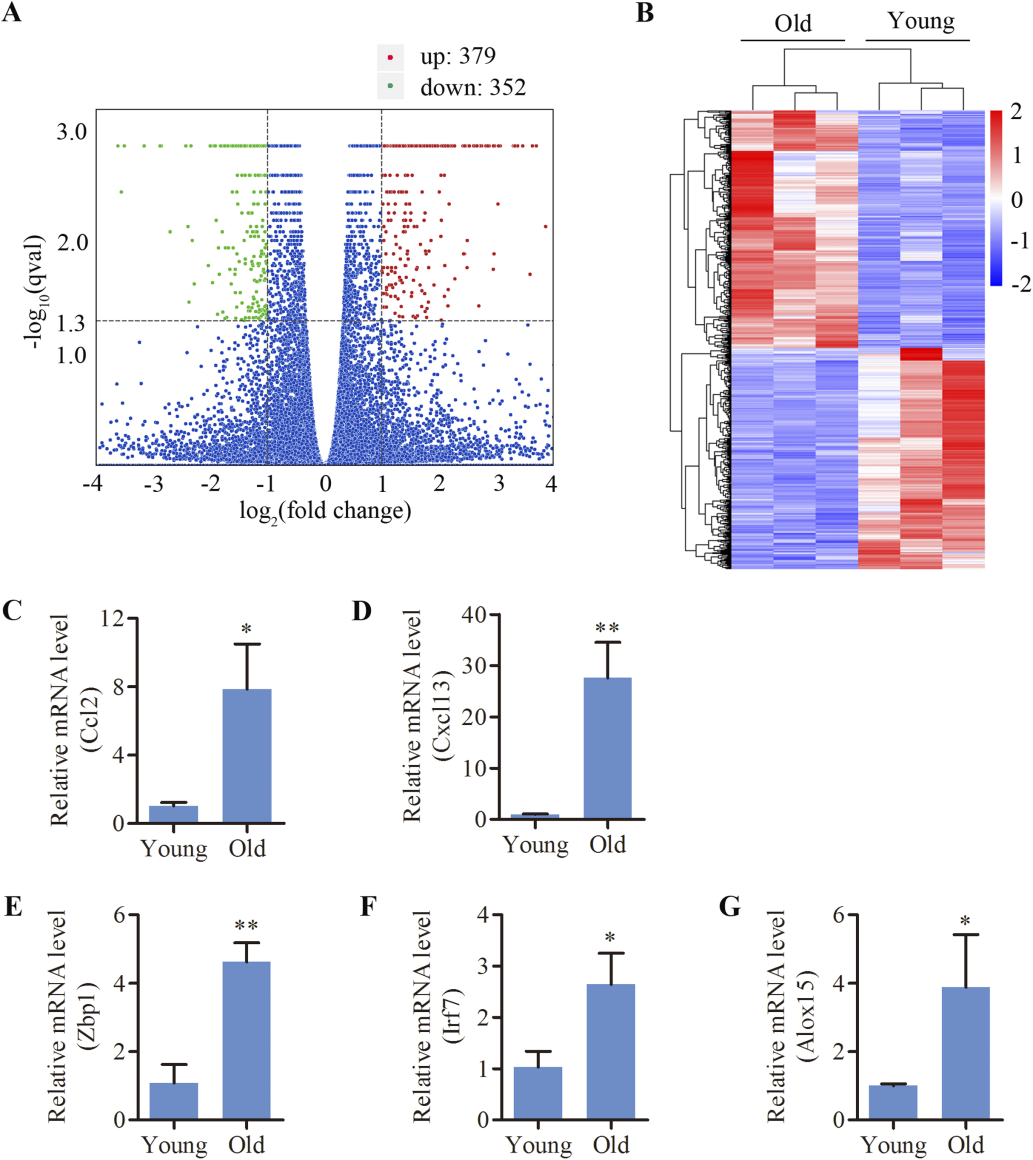
Differentially expressed mRNAs in the cochlea of aged C57BL/6 mice. (A) Volcano plot showing comparative mRNA expression in the cochlea of the young and old C57BL/6 mice (*n* = 3). Red dots represent the significantly upregulated transcripts (379) and green dots indicate the significantly downregulated transcripts (352) in old mice (*p*-adjust < 0.05). Blue dots: no significant difference. (B) Heat map showing hierarchical clustering of differentially expressed mRNAs. Red indicates upregulation and blue representes downregulation. (C–G) Validation of five differentially expressed mRNAs by real-time qPCR. Bars show mean ± SD. *p < 0.05, **p < 0.01.

C57BL/6 mice, a mouse model of early-onset AHL, were used in this study. We analyzed the cochlear DEGs of 12-month mice compared to 4-week old mice. A total of 731 genes (379 up- and 352 down-regulated) were revealed to be differentially expressed in the cochlea of aged mice compared to the young (Figs. 1A and 1B). Lists of the 20 most upregulated and downregulated genes were shown in Tables 1 and 2, respectively. Using the GenAge database (http://genomics.senescence.info/genes/), aging-related genes such as *IL7R*, *FOS*, *C1QA*, *EGR1*, *CCN2*, *SNCG*, *UCHL1*, *KL*, *ARNTL* and *NRG1* were found to be significantly modulated in the aged cochlea (Table 3). Moreover, multiple genes related to apoptosis and necroptosis such as *FOS*, *CASP4*, *GADD45G*, *XAF1*, *NR4A1*, *DDIT4*, *GDF15*, *TNFRSF12A*, *EGR1*, *SLC5A11*, *PARP3*, *ANXA1*, *HK2*, *GADD45B*, *CTSC* and *MLKL* were significantly upregulated in the aged mice (Table 4). Interestingly, we identified 232 genes (31.7% of the total DEGs) are associated with immune responses and inflammation in the aged cochlea by referring to a list of immunity-related genes mentioned in a previous study (Kim et al., 2019a). Aged mice showed increased expressions of multiple complement system-related genes such as *C1QA*, *C1QB* (log 2 fold change 1.743), *C1QC* (log 2 fold change 1.909), *C1RA* (log 2 fold change 1.184), *C4B* (log 2 fold change 2.237) and *C3* (log 2 fold change 2.157). In addition, molecular markers specific and/or consistent with the presence of macrophages were found to be significantly increased in the aged cochlea (Table 5). Inflammation-related genes (*CXCL13, ZBP1, IRF7, CCL2, ALOX15*), which among the top 20 upregulated transcripts, were selected for qPCR verification. The selected genes displayed the same expression pattern with the RNA-sequencing data (Figs. 1C–1G).

**Table 1:** Top 20 upregulated DEGs in the aged cochlea compared to the young cochlea.

No.	Gene	Description	log 2 fold change
1	Cd300ld3	CD300 molecule like family member D3	Infinity
2	Cxcl13	Chemokine (C–X–C motif) ligand 13	4.838
3	F830016B08Rik	RIKEN cDNA F830016B08 gene	4.735
4	Ms4a4c	Membrane-spanning 4-domains, subfamily A, member 4C	4.489
5	Muc5b	Mucin 5, subtype B, tracheobronchial	4.401
6	Mx1	MX dynamin-like GTPase 1	4.248
7	Zbp1	Z-DNA binding protein 1	4.037
8	Itgax	Integrin alpha X	3.867
9	Irf7	Interferon regulatory factor 7	3.705
10	Serpina3n	Serine (or cysteine) peptidase inhibitor, clade A, member 3N	3.636
11	Ccl2	Chemokine (C–C motif) ligand 2	3.595
12	Gbp10	Guanylate-binding protein 10	3.467
13	Tgtp1	T cell specific GTPase 1	3.377
14	Siglec1	Sialic acid binding Ig-like lectin 1	3.364
15	H2-Q6	Histocompatibility 2, Q region locus 6	3.328
16	Gm4951	Predicted gene 4951	3.322
17	Ifi44	Interferon-induced protein 44	3.303
18	Oas1g	2′–5′ Oligoadenylate synthetase 1G	3.073
19	Gm4841	Predicted gene 4841	3.045
20	Alox15	Arachidonate 15-lipoxygenase	3.034

**Table 2:** Top 20 downregulated DEGs in the aged cochlea compared to the young cochlea.

No.	Gene	Description	log 2 fold change
1	Bglap	Bone gamma carboxyglutamate protein	−3.618
2	St6galnac6	ST6 *N*-acetylgalactosaminide alpha-2,6-sialyltransferase 6	−3.557
3	Col1a1	Collagen, type I, alpha 1	−3.505
4	Slc4a1	Solute carrier family 4 member 1	−3.157
5	Ppp1r26	Protein phosphatase 1, regulatory subunit 26	−2.879
6	Ibsp	Integrin binding sialoprotein	−2.858
7	Gypa	Glycophorin A	−2.703
8	Col3a1	Collagen, type III, alpha 1	−2.427
9	Gpr165	G protein-coupled receptor 165	−2.390
10	Myl1	Myosin, light polypeptide 1	−2.372
11	Bpifa1	BPI fold containing family A, member 1	−2.338
12	Nrg1	Neuregulin 1	−2.316
13	Bves	Blood vessel epicardial substance	−2.281
14	Mmp8	Matrix metallopeptidase 8	−2.034
15	Mepe	Matrix extracellular phosphoglycoprotein	−2.007
16	Galnt13	Polypeptide *N*-acetylgalactosaminyltransferase 13	−2.002
17	Col1a2	Collagen, type I, alpha 2	−1.965
18	Hapln1	Hyaluronan and proteoglycan link protein 1	−1.963
19	Kif21a	Kinesin family member 21A	−1.961
20	S100a8	S100 calcium binding protein A8	−1.942

**Table 3:** Genes related to aging.

No.	Gene	Description	log 2 fold change
1	Il7r	Interleukin 7 receptor	2.607
2	Fos	FBJ osteosarcoma oncogene	1.955
3	C1qa	Complement component 1, q subcomponent, alpha polypeptide	1.893
4	Egr1	Early growth response 1	1.419
5	Ccn2	Cellular communication network factor 2	1.158
6	Sncg	Synuclein gamma	1.009
7	Uchl1	Ubiquitin carboxy-terminal hydrolase L1	−1.126
8	Kl	Klotho	−1.182
9	Arntl	Aryl hydrocarbon receptor nuclear translocator like	−1.220
10	Nrg1	Neuregulin 1	−2.316

**Table 4:** Genes related to apoptosis and necroptosis.

No.	Gene	Description	log 2 fold change
1	Fos	FBJ osteosarcoma oncogene	1.955
2	Casp4	Caspase 4	1.862
3	Gadd45g	Growth arrest and DNA damage inducible gamma	1.838
4	Xaf1	XIAP associated factor 1	1.758
5	Nr4a1	Nuclear receptor subfamily 4 group a member 1	1.567
6	Ddit4	DNA damage inducible transcript 4	1.569
7	Gdf15	Growth differentiation factor 15	1.520
8	Tnfrsf12a	TNF receptor superfamily member 12a	1.435
9	Egr1	Early growth response 1	1.419
10	Slc5a11	Solute carrier family 5 member 11	1.406
11	Parp3	Poly(ADP-ribose) polymerase family member 3	1.160
12	Anxa1	Annexin a1	1.137
13	Hk2	Hexokinase 2	1.082
14	Gadd45b	Growth arrest and DNA damage inducible beta	1.050
15	Ctsc	Cathepsin c	1.042
16	Mlkl	Mixed lineage kinase domain like pseudokinase	1.031

**Table 5:** Markers specific and/or consistent with the presence of macrophages/dendritic cells.

No.	Gene	Description	log 2 fold change
1	Ptprc	Protein tyrosine phosphatase, receptor type, C	1.408
2	Cd68	CD68 antigen	1.822
3	Cd14	CD14 antigen	1.799
4	H2-Aa	Histocompatibility 2, class II antigen A, alpha	2.822
5	H2-Ab1	Histocompatibility 2, class II antigen A, beta 1	2.977
6	H2-Eb1	Histocompatibility 2, class II antigen E beta	2.718
7	Lgals3	Lectin, galactose binding, soluble 3	2.526
8	Itgax	Integrin alpha X	3.867
9	H2-K1	Histocompatibility 2, K1, K region	1.589
10	C1ra	Complement component 1, r subcomponent A	1.184
11	C1qa	Complement component 1, q subcomponent, alpha polypeptide	1.893
12	C1qb	Complement component 1, q subcomponent, beta polypeptide	1.743
13	C3	Complement component 3	2.157
14	C4b	Complement component 4	2.237
15	Ctss	Cathepsin S	2.115
16	Mpeg1	Macrophage expressed gene 1	2.048
17	Fcgr1	Fc receptor, IgG, high affinity I	1.975
18	Fcgr2b	Fc receptor, IgG, low affinity IIb	1.107

### GO and KEGG enrichment analysis of the differentially expressed genes

**Figure 2:**
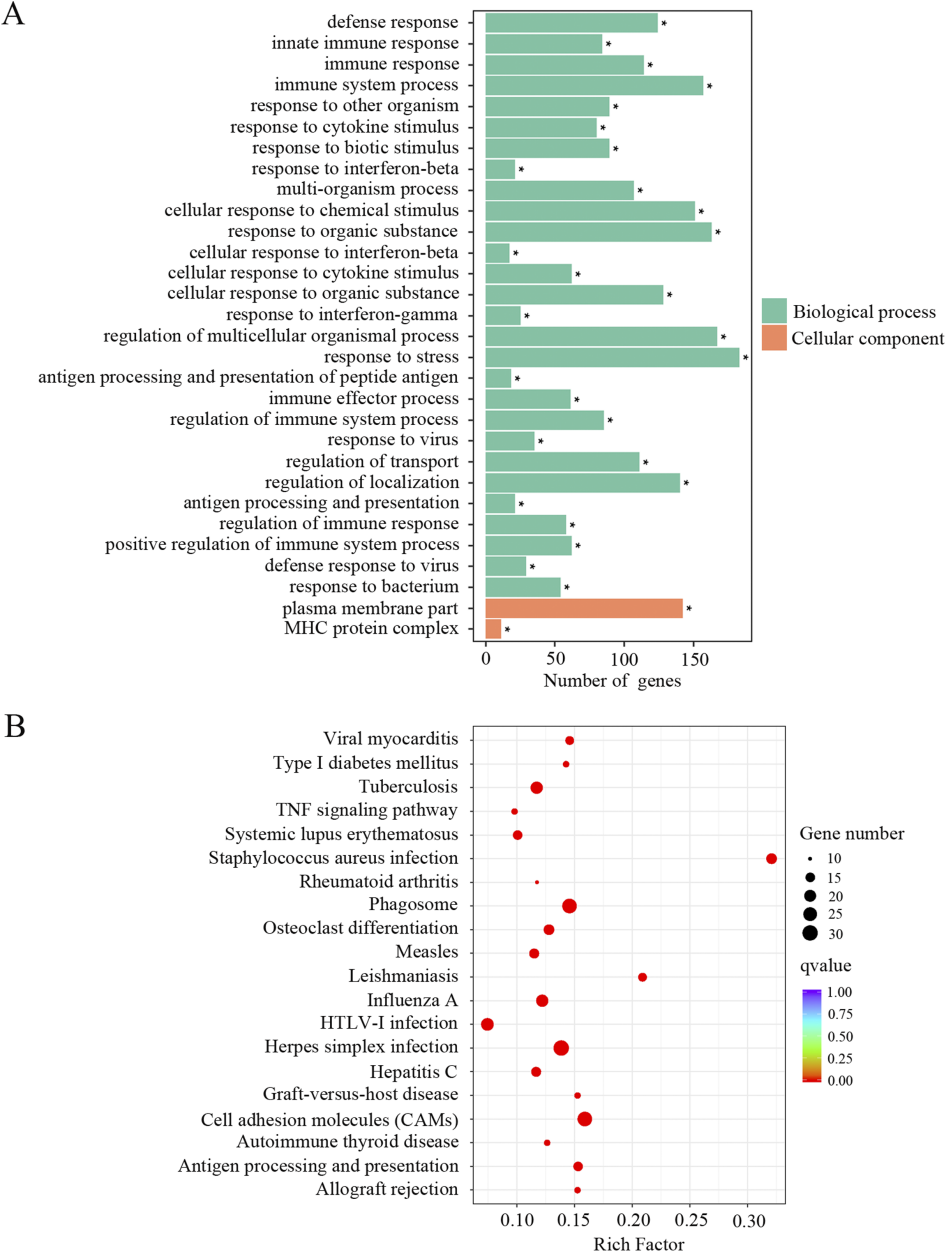
GO and KEGG analysis of the total differentially expressed genes. (A) GO enrichment analysis of the total DEGs. *p*-adjust < 0.05. (B) KEGG enrichment analysis of the total DEGs. *p*-adjust < 0.05.

In order to identify the relevant biological functions and enriched signaling pathways of the DEGs, GO and KEGG analyses were performed. In total, 522 GO terms were significantly enriched in the comparison of old and young mice. As shown in Fig. 2A and Supplemental Material 1, the most enriched top 10 GO terms included defense response, innate immune response, immune response, immune system process, response to other organism, response to cytokine stimulus, response to biotic stimulus, response to interferon-beta, multi-organism process and cellular response to chemical stimulus. KEGG pathway enrichment analysis revealed 66 significantly enriched terms. As shown in Fig. 2B and Supplemental Material 1, the most enriched top 10 KEGG terms included herpes simplex infection, cell adhesion molecules, phagosome, staphylococcus aureus infection, influenza A, tuberculosis, leishmaniasis, osteoclast differentiation, antigen processing and presentation and viral myocarditis.

### Up-regulation of genes associated with immune responses and inflammatory pathways

**Figure 3:**
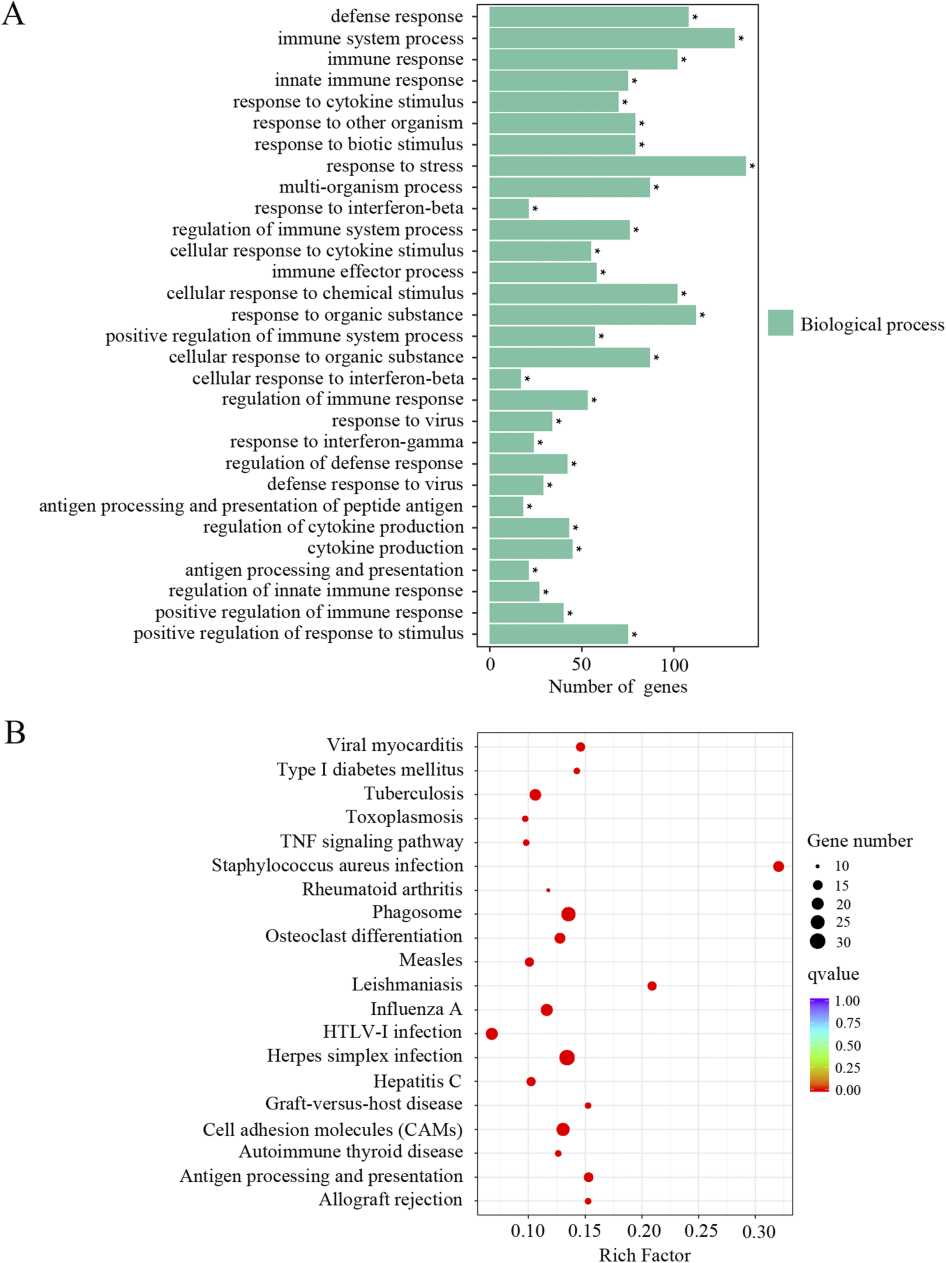
GO and KEGG analysis of the upregulated DEGs. (A) GO enrichment analysis of the upregulated DEGs. *p*-adjust < 0.05. (B) KEGG enrichment analysis of the upregulated DEGs. *p*-adjust < 0.05.

The upregulated DEGs were separated for GO and KEGG enrichment analysis. In total, 521 GO terms were significantly enriched in aged cochlea compared to the young. Among them, immune responses and inflammatory pathways were the most prominent GO terms. As shown in Fig. 3A and Supplemental Material 1, plenty of such GO terms include but not restricted to defense response, immune system process, immune response, innate immune response, regulation of immune system process, immune effector process, positive regulation of immune system process, regulation of immune response, regulation of defense response and antigen processing and presentation of peptide antigen. Besides, GO terms related to responses to various stresses were also significantly affected in aged mice. It is noteworthy that GO terms related to macrophage activation were significantly enriched in aged mice, such as macrophage activation and monocyte chemotaxis. Interestingly, the GO term “aging” and terms related to cell death were found significant as well. KEGG pathway analysis revealed 66 terms significantly enriched in aged mice compared to the young. As shown in Fig. 3B and Supplemental Material 1, KEGG terms correlated with infectious and immune diseases, immune responses and inflammatory pathways were significantly enriched. Multiple number of such terms include but not limited to herpes simplex infection, type I diabetes mellitus, autoimmune thyroid disease, phagosome, cell adhesion molecules, antigen processing and presentation, complement and coagulation cascades, TNF signaling pathway, toll-like receptor signaling pathway, Jak-STAT signaling pathway and NF-κB signaling pathway. KEGG analysis also showed the term “apoptosis” in aged mice compared to the young. These data revealed an intense correlation of aging cochlea with immunity and inflammation compared to the young.

### Down-regulation of genes associated with ion channel fucnction and neuronal signaling

**Figure 4:**
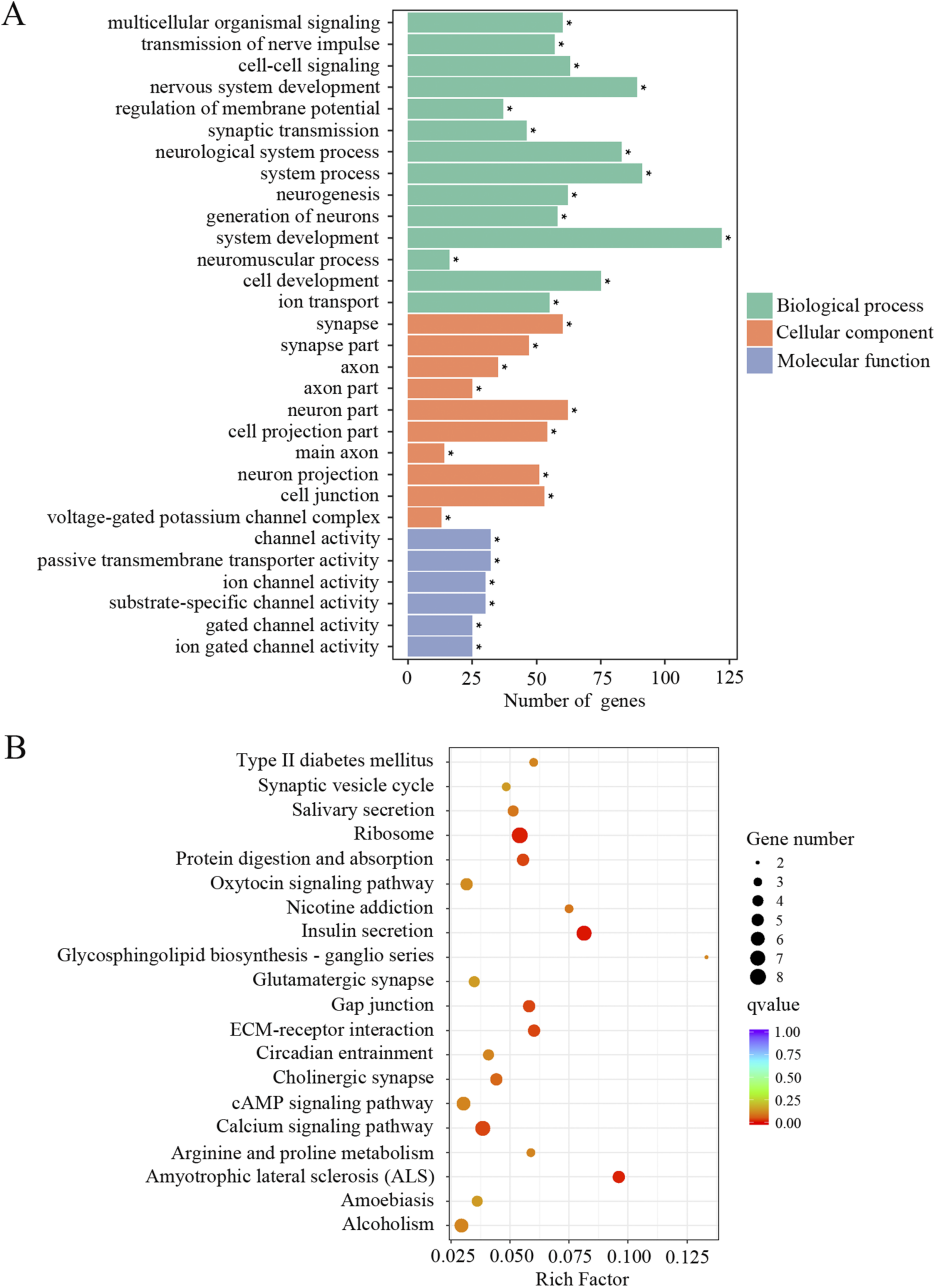
GO and KEGG analysis of the downregulated DEGs. (A) GO enrichment analysis of the downregulated DEGs. *p*-adjust < 0.05. (B) KEGG enrichment analysis of the downregulated DEGs. *p* < 0.05.

The downregulated transcripts were also separated for GO and KEGG enrichment analysis. In total, 186 GO terms were found significantly enriched in the aged mice compared to the young mice. Among the list of GO terms, ion channel function and neuronal signaling were the most enriched GO terms. As shown in Fig. 4A and Supplemental Material 1, numerous such GO terms include but not restricted to transmission of nerve impulse, cell–cell signaling, regulation of membrane potential, synaptic transmission, channel activity, passive transmembrane transporter activity, ion channel activity, substrate-specific channel activity, voltage-gated potassium channel complex, gated channel activity, ion gated channel activity and ion transport. KEGG pathway analysis also revealed pathways involved in ion channel function and neuronal signaling, such as calcium signaling pathway, cholinergic synapse, synaptic vesicle cycle and glutamatergic synapse (Fig. 4B; Supplemental Material 1). These data suggested that ion channel function and neuronal transmission declined in the cochlea with age.

### Oxidative stress induced the expression of inflammation and necroptosis-related genes in HEI-OC1 cells

**Figure 5:**
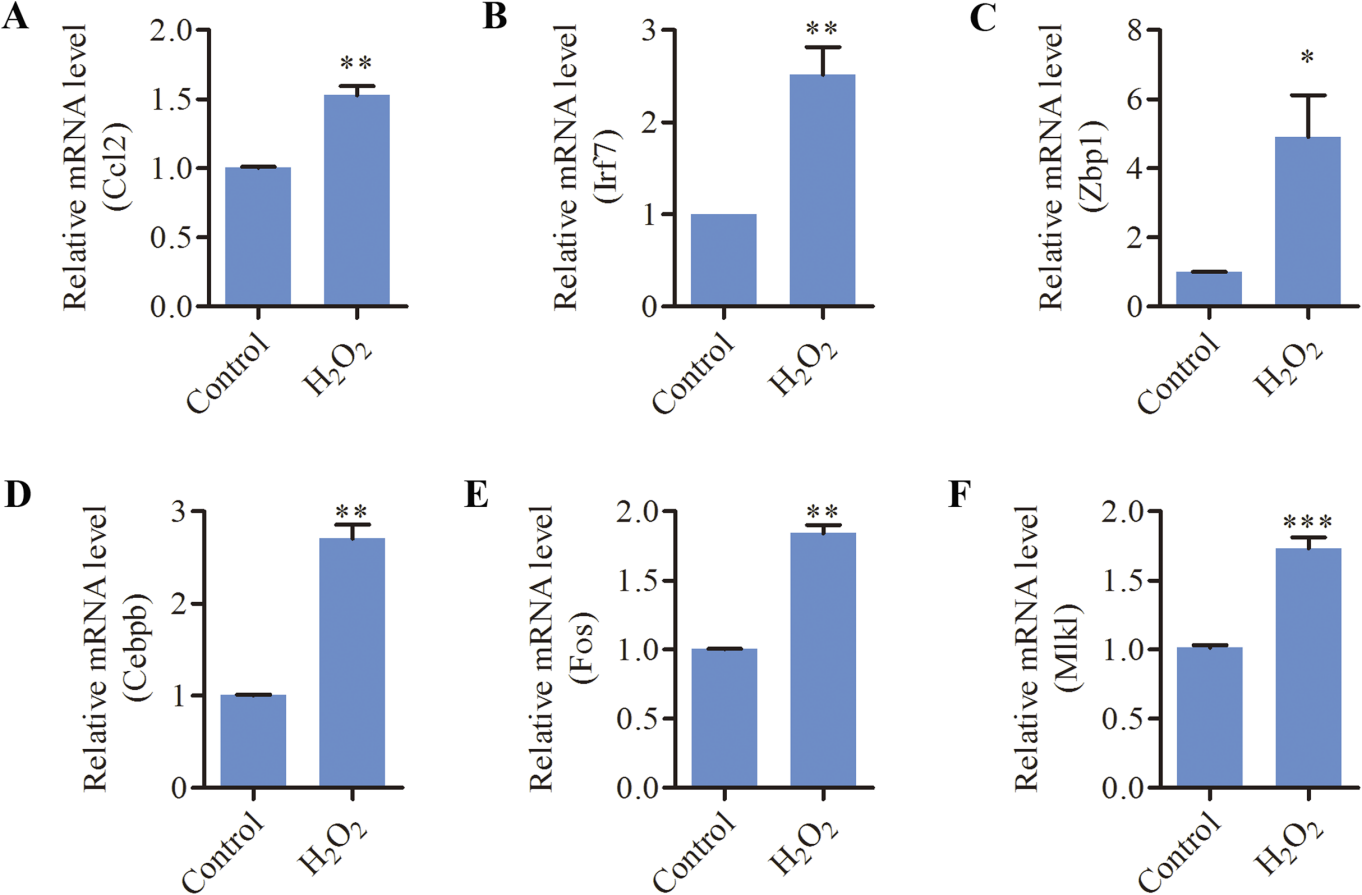
H_2_O_2_ induced inflammation and necroptosis in HEI-OC1 cells. HEI-OC1 cells were treated with 500 μM H_2_O_2_ for 6 h. (A–F) Real-time qPCR analysis showed that H_2_O_2_ treatment increased the expression of *CCL2, IRF7, ZBP1, CEBPB, FOS* and *MLKL*. Bars show mean ± SD. *p < 0.05, **p < 0.01, ***p < 0.001.

Since oxidative stress has been implicated as a causative factor for AHL, we tested the effects of oxidative stress on inflammation and necroptosis in HEI-OC1 cells. As shown in Fig. 5, the mRNA levels of inflammation-related genes (*CCL2, ZBP1, IRF7, CEBPB, FOS*) and necroptosis-related genes (*MLKL, ZBP1*) were significantly increased in response to H_2_O_2_ treatment. These data suggested that oxidative stress induced inflammation and necroptosis in HEI-OC1 cells.

## Discussion

The current study was designed to determine the transcriptional changes of cochlear genes and the most significantly affected functions and pathways during aging in C57BL/6 mice using next generation sequencing. Our RNA-sequencing data revealed that transcripts associated with aging, apoptosis and necroptosis were significantly modulated in aged cochleae. Importantly, numerous genes related to immune responses and inflammation were differentially expressed during aging. Bioinformatics analysis of the upregulated DEGs also revealed that a large portion of biological processes and pathways are related to immune and inflammatory pathways, such as complement system and macrophage activation. Whereas, lots of the downregulated genes are involved in biological processes and pathways associated with ion channel function and neuronal signaling. In HEI-OC1 cells, H_2_O_2_ treatment significantly induced the expressions of multiple genes related to inflammation and necroptosis.

In our RNA-sequencing data, lots of aging-related genes such as *KL, NRG1, FOS, EGR1, UCHL1, C1QA, IL7R, ARNTL, CTGF* and *SNCG* were found differentially expressed with age. Aging-suppressor gene KL regulates multiple growth factor signaling pathways and response to oxidative stress (Kuro-o, 2008), and has been reported to be a key mediator of auditory function (Kamemori et al., 2002). In this study, a significant decrease in *KL* was observed in the cochlea of aged mice, which is consistent with the previous study (Takumida et al., 2009). In our study, a significant reduction of *NRG1*, a direct modulator of synaptic transmission, was identified in the aged mice, which could impair the synaptic transmission between spiral ganglion neurons (SGNs) and inner hair cells (IHCs) (Jin et al., 2011). A previous study revealed that noise exposure induced the expression of FOS, EGR1 in rat cochlea, which may be associated with cochlear damage (Cho et al., 2004). Deficit in UCHL1 expression was reported to promote gentamicin-induced ototoxicity probably via impairing autophagic flux in rat cochlear organotypic cultures (Kim et al., 2019b). It is important for future investigation to establish the roles of these aging-related transcripts in the process of cochlear degeneration with age.

In our study, multiple apoptosis-related genes were markedly induced with aging, indicating apoptosis signaling activation in aged cochleae. Notably, the necroptosis marker *MLKL* was significantly upregulated in the cochlea of aged mice. Moreover, our study also revealed a marked increase in the expression of *ZBP1*, an important gene involved in necroptosis activation (Grootjans, Vanden & Vandenabeele, 2017). These findings suggested, in addition to apoptosis, necroptosis may be another possible route of cell death in aged cochleae. Necroptosis, a new form of regulated cell death, can result in the release of damage-associated molecular patterns (DAMPs), which further initiate inflammatory responses (Royce, Brown-Borg & Deepa, 2019). Necroptosis-mediated inflammation has been demonstrated to play a vital role in multiple age-associated disorders such as Alzheimer’s disease, Parkinson’s disease and atherosclerosis (Royce, Brown-Borg & Deepa, 2019). Several studies revealed that necroptosis contributed to drug- and noise-induced hearing loss (Choi et al., 2019; Ruhl et al., 2019; Zheng, Chen & Sha, 2014). In this study, oxidative stress significantly induced the expression of *MLKL*, *ZBP1* and some inflammation-related genes in HEI-OC1 cells, which is similar to our observation in the mouse model. The effects of necroptosis-mediated inflammation on age-related cochlear degeneration need to be further investigated in our future study.

Increasing evidence has demonstrated a key role of inflammation in the development of aging-related diseases such as neurodegeneration disease, cardiovascular disease and diabetes (Pawelec, Goldeck & Derhovanessian, 2014; Chen & Xu, 2015). However, the effect of inflammation on cochlear degeneration during aging is still largely unknown. Our RNA-sequencing data revealed an interesting discovery that numerous genes related to immune and inflammatory responses are significantly modulated in the cochleae of aged mice. Bioinformatics analysis of the upregulated DEGs further uncovered multiple processes and pathways are associated with immune and inflammatory responses. These findings suggest chronic inflammation may be associated with aging-related cochlear degeneration. In this study, the terms TNF signaling pathway, toll-like receptor signaling pathway, Jak-STAT signaling pathway and NF-κB signaling pathway were found significantly enriched for upregulated genes by KEGG analysis. These signalings may regulate the process of inflammation in the aged cochlea. The complement system is an important component of the immunity system, and chronic complement activation has been supposed to be associated with glial activation, and synapse and neuron loss in the aging central nervous system (Lee, Coulthard & Woodruff, 2019). Significant upregulation of complement proteins CFI and C1S was observed in noise-traumatized rat cochleae (Patel et al., 2013). Similarly, our data identified multiple complement system-related genes such as *C1QA, C1QB, C1QC, C1RA, C4B* and *C3* upregulated in the cochlea of aged mice. Complement system may be involved in the cochlea responses to acoustic trauma and aging. Immune cells such as macrophages are believed to contribute to the onset and progression of aging-associated degenerative diseases (Latta, Brothers & Wilcock, 2015). SAMP8 mice display premature cochlear degeneration and were found elevated number of CD45-positive macrophage in aged cochleae (Menardo et al., 2012). Moreover, increased population of activated macrophages in the auditory nerve with age was observed in the human cochleae (Noble et al., 2019). Activation of macrophage was reported to precede sensory cell pathogenesis in aging mouse cochleae (Frye et al., 2017). Our data revealed that markers for macrophages were upregulated in the aged cochleae, suggesting increased macrophage numbers with aging. Meanwhile, *CCL2* and *CCL5*, two important chemokines involved in monocyte/macrophage migration and infiltration, were found to be markedly increased in the aged cochleae. In addition, the GO term macrophage activation was significantly enriched for upregulated genes *TLR2, TLR7, TYROBP, LGALS9, AIF1, SLC11A1* and *MUC5B*. These genes may participate in the activation of macrophages in the cochlea of aged mice. Together, immune and inflammatory responses including complement system and macrophage activation were significantly induced in the cochleae during aging, suggesting the possible involvement of chronic inflammation in age-related cochlear degeneration.

Cochlear ion channels play a critical role in maintaining normal hearing. They are crucial for supporting hair cell development, maintaining the endocochlear potential (EP) and synaptic transmission (Tawfik et al., 2019; Fuchs, 1996). Many studies have identified plenty of mutations in membrane transport proteins which result in progressive hearing loss with age (Tawfik et al., 2019). Reduced ion channel levels with age have been observed in animal models. The Na, K-ATPase ion channel in the cochlear lateral wall, important for maintaining EP, was reported to decline with age in murine models (Schulte & Schmiedt, 1992; Ding et al., 2018). In this study, multiple potassium channel associated genes (such as *KCNAB2, KCNC1, KCNIP1, KCNJ11, KCNJ2, KCNQ3* and *KCNS1*), calcium channel associated genes (such as *CACNA1E* and *CACNG2*) and sodium channel associated genes (such as *SCN1A*) showed reduced expression in aged cochlea. Bioinformatics analysis of the downregulated DEGs revealed plenty of the most significantly affected functions and pathways are involved in ion channel function and neuronal signaling. These alterations may impair the EP and the synaptic transmission between HCs and SGNs in the cochlea. Synaptic transmission in the central auditory system also deteriorates during aging. Xie & Manis (2017) reported that synaptic transmission between SGNs and bushy neurons of the cochlear nucleus is degraded in aged mice.

A previous study reported changes of gene expression during aging in the cochlea using microarray technology (Marano, Tickner & Redmond, 2012). There are some differences in the identified DEGs between our study and theirs. The discrepancy could result from differences in the experimental paradigms, such as sample composition of the cochlear tissue, amount of each sample and technology used for genetic test. Our study provides some intresting findings, such as cochlear inflammation and necroptosis, that may help us further understand the molecular mechanism of cochlear degeneration with age. However, further investigations are needed to confirm the roles of the transcripts and pathways identified in this study in the development of AHL.

## Conclusion

In summary, using next generation sequencing, our transcriptomic analysis revealed the differences of gene expression pattern with age in the cochlea of C57BL/6 mice. Many DEGs related to aging, apoptosis, necroptosis and ion channels were identified. Our study also revealed multiple immune and inflammatory transcriptomic changes during cochlear aging and provides new insights into the molecular mechanisms underlying cochlear inflammation in AHL.

## Supplemental Information

10.7717/peerj.9737/supp-1 Supplemental Information 1 GO and KEGG enrichment analysis of the DEGs.

10.7717/peerj.9737/supp-2 Supplemental Information 2 Raw data for PCR.

## Additional Information and Declarations

### Data Availability

The following information was supplied regarding data availability: The sequencing data is available at NCBI GEO: GSE127204. Data, code, and visualization through dedicated Shiny App are available at GitHub: https://github.com/microgenomics/HumanMicrobiomeAnalysis.
